# Contact dynamics investigation towards microgravity experiment for asteroid-related scenarios

**DOI:** 10.1007/s11044-025-10091-z

**Published:** 2025-11-25

**Authors:** Samuele Vaghi, Iosto Fodde, Paolo Panicucci, Alessia Cremasco, Fabio Ferrari

**Affiliations:** Department of Aerospace Science and Technology, Politecnico di Milano, Via La Masa 34, Milan, 20156 Milan Italy

**Keywords:** Contact dynamics, State estimation, Granular mechanics, N-body codes

## Abstract

Most asteroids are now considered gravitational aggregates of loosely consolidated material, and their granular nature suggests that their dynamics can be effectively simulated using N-body codes, such as GRAINS, whose contact dynamics engine is based on the open source code Chrono. Currently, contact parameters in N-body codes are usually tuned to reproduce the macroscopic behavior of large-scale scenarios. Recent in-situ measurements revealed unexpected behavior of granular media on asteroid surfaces, exposing the limitations of current scaling models. Accurately modeling the interactions at particle scale is key to enhance the realism of the models and simulations necessary to support future asteroid exploration missions. This work presents the set-up of an experimental campaign for investigating contact physics in asteroid-related scenarios. The outcome of the campaign can be then used for fine tuning and calibration of contact parameters in multi-body codes. The high-level goal of the experiment is to observe the collision between two asteroid simulant cobbles in micro-gravity and vacuum conditions. Then, a digital twin of the experiment will be calibrated to reproduce their 6-dof trajectory to the best accuracy possible. This work describes the experiment’s design and preliminary testing, including requirements and constraints identified for each component of the experimental set up, focusing on the benchmark model of the digital twin and on the strategy designed to estimate the cobbles’ states and contact parameters. The estimation results from preliminary numerical simulations show good performance in all the scenarios tested, providing important guidelines for the next phases of the experiment development.

## Introduction

In recent years, there has been a growing interest in asteroid exploration and research. From a scientific perspective, asteroids provide unique information about the early ages of the Solar System [1]. Moreover, there is evidence of the presence of huge amounts of metals and Earth-rare materials on asteroids. Therefore, their scientific interest is complemented by their economical potential for resource utilization [1]. Asteroids also represent a threat to life on Earth; therefore, governments and space agencies are investing in planetary defense missions, such as NASA-DART [2]. Launched on 24 November 2021, DART successfully altered the orbit of asteroid Dimorphos on 26 September 2022, demonstrating the viability of the kinetic impact technique for planetary defense [3].

It is now believed that most Near-Earth asteroids with diameter larger than 100 m are rubble-piles. Rubble-piles are aggregates of loose material bound together by self-gravity, rather than by the strength of their bulk material [4]. To study their evolution through numerical simulations, it is necessary to solve a gravitational-collision problem. Recent studies have demonstrated that complex evolution scenarios can be effectively modeled through N-body codes [5–7], which also incorporate the collision dynamics of the rigid bodies. In general, Discrete Element Methods (DEM) are employed to introduce contact dynamics in gravitational N-body codes involving low-energy collisions [8]. These methods can be classified into two categories: soft-body and hard-body contact methods. In soft-contact methods, the bodies are allowed to experience a small overlap before the contact force is introduced [9]. The contact forces are modeled according to a spring-dashpot system. In contrast, hard-body contact methods are impulse-based methods that rely solely on the coefficient of restitution (CoR) to describe the contact [10]. Applications of both soft and hard contact methods to granular material in the asteroid environment can be found in literature [5, 11]. A key requirement for N-body simulations is the ability to handle irregularly-shaped particles. Studies have shown that angular particles are crucial for representing interlocking effects in gravitational aggregates, which enhances the realism of the simulations [12, 13]. The N-body code GRAINS, whose contact dynamics is based on the multi-body code Chrono (C::E) [14, 15], has proven able to reproduce relevant asteroid evolution scenarios, including non-spherical particles in the simulation [6, 16]. The contact dynamics libraries from C::E enable the implementation of both soft and hard contact methods.

The reliability of C::E in modeling granular materials has been evaluated through both numerical and experimental tests in various studies, including those focused on asteroid-related scenarios [17]. However, validating the contact dynamics at the level of local interactions between irregularly-shaped particles remains an open challenge. Whether using soft or hard contact models, several parameters, such as friction and the coefficient of restitution, influence the simulations. These parameters are typically tuned to represent full-scale scenarios rather than the contact dynamics at local scale. It is expected that a code calibrated to reliably represent the contact dynamics at particle scale will lead to more realistic simulations. An experimental campaign is being developed to fill this gap. The primary objective is to observe the collision between two asteroid simulant cobbles under microgravity and vacuum conditions and to reconstruct their six degrees of freedom (6-DoF) trajectory. The shape of each cobble will be acquired using a 3D scanner to create a high-fidelity digital twin in GRAINS. Leveraging the results from the microgravity experiment, the simulation code will be calibrated to accurately reproduce the post-impact trajectory.

This paper aims to present the approach and methodologies implemented for the design, testing and realization of the experimental campaign, as well as to discuss the lessons learned and preliminary results obtained during the preparation phase.

The paper is organized as follows: Sect. 2 describes the rigid body model used as benchmark for the experiment’s digital twin. Section 3 a provides a detailed overview of the hardware requirements necessary to meet the experiment’s high-level objectives, while also considering the constraints imposed by the facility. Additionally, the estimation strategy is outlined. In Sect. 4 results from numerical simulations about the state and contact parameters estimation are discussed. These will serve as guidelines for the further phases of experiment development. Final remarks are discussed in Sect. 5.

## Dynamics

The primary objective of the experiment is to characterize particle-scale interactions in a simulated asteroid environment (i.e. micro-gravity and vacuum), which can then be used to validate numerical simulations in N-body codes, such as GRAINS. To validate the methodology, a digital twin of the bodies has been developed using C::E. Once data from multiple microgravity launches are collected, a specialized high-fidelity model will be constructed to replicate each individual collision event.

**Fig. 1:**
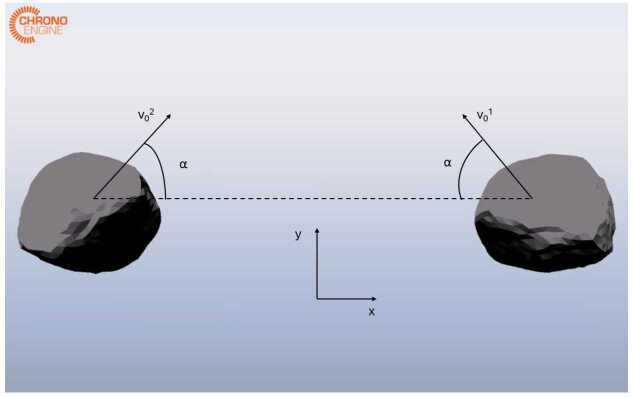
Snapshot of the colliding bodies in the numerical simulation

Asteroid-simulant cobbles can be treated as three-dimensional rigid bodies, with both translational and rotational degrees of freedom. Hence, their state can be expressed through the vector $\boldsymbol{x} = [\boldsymbol{r}_{CoM}, \boldsymbol{v}_{CoM}, \boldsymbol{q}_{NB}, {^{\textit{B}}} \boldsymbol{\omega}_{NB}]^{\top}$, where $\boldsymbol{r}_{CoM}$ and $\boldsymbol{v}_{CoM}$ are the position and velocity of the body’s Center of Mass (CoM) respectively, $\boldsymbol{q}_{NB}$ is the quaternion expressing the attitude of the body’s principal axis frame with respect to the inertial frame, and ${^{\textit{B}}}\boldsymbol{\omega}_{NB}$ is the angular velocity from the cobble’s body frame to the inertial frame, expressed in body frame. Colliding bodies for preliminary tests are created using the shape model of the Bennu asteroid, based on radar observations [18]. This has been selected as it presents surface irregularities that are commonly found on asteroid material, and is available as a high-resolution mesh. The mesh has been slightly modified in Blender to adapt it to the simulation environment. Its inertial properties and dimensions are reported in Table 1, and Fig. 1 shows the colliding bodies.

**Table 1:** Properties of Bennu’s shape model

Mass [*kg*]	Characteristic lenght [*cm*]	Inertia tensor *I* [$kg/m^{2}$]	N. vertices
1.1571	11.88	diag([0.001119,0.001010,0.000817])	1348

This shape represents a balanced compromise between simulation realism and computational efficiency. The number of vertices allows for the creation of a complex collision scenario in C::E, while avoiding memory issues on a standard laptop. Indeed, the memory footprint calculated for a simulation using the mesh reported in Table 1 is 281 MB. A comparison has been performed using the same shape with 193824 vertices: the resulting memory footprint is 10445 MB. These data refer to a machine mounting an Intel i7-13700H processor.

**Fig. 2:**
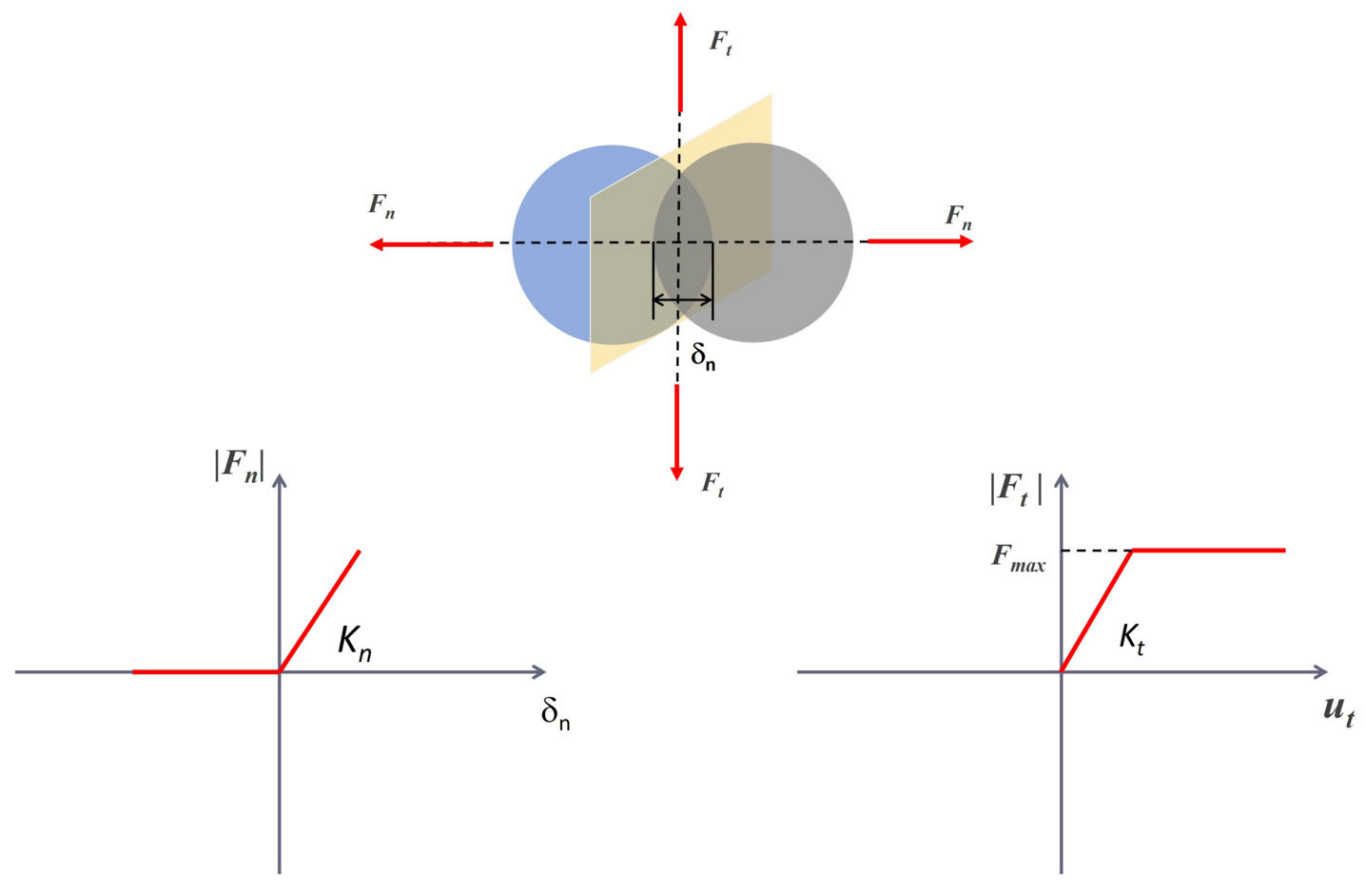
Smooth Force Model, considering a Hookean contact law. Adapted from [9]

**Fig. 3:**
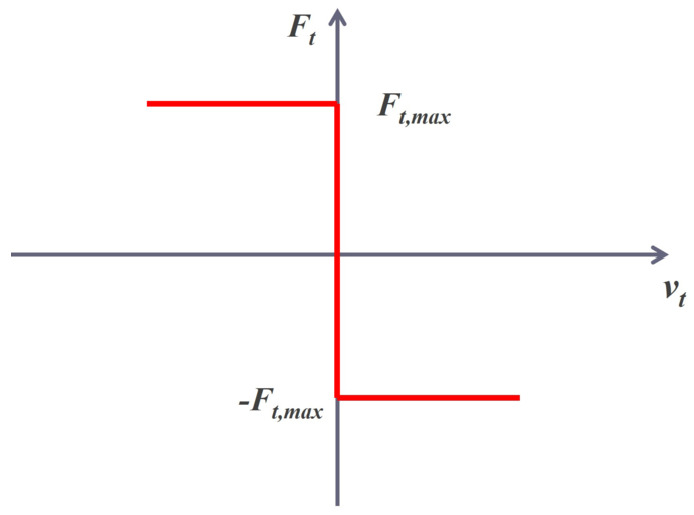
Non Smooth model. $v_{t}$ is the tangential velocity between the bodies

As previously mentioned, two different contact models can be used to simulate the collision. Both models fall under the category of Discrete Element Methods (DEM). In soft-body (or smooth) methods, the discontinuity introduced by the contact is *regularized*, such that ordinary time steppers can be used to integrate the system. The colliding bodies are allowed to experience a small overlap before a corrective force is introduced [9]. The normal and tangential forces, $F_{n}$ and $F_{t}$, are computed based on a contact constitutive law that is based on a spring-mass-damper representation:

1 $$\begin{aligned} F_{n} = f(\bar{R}, \delta _{n}) (K_{n} u_{n} - C_{n} \bar{m} v_{n}) \\ F_{t} = f(\bar{R}, \delta _{n}) (-K_{t} u_{t} - C_{t} \bar{m} v_{t}) \end{aligned}$$

where $\bar{R}$ and $\bar{m}$ are the effective radius of curvature and mass of the bodies, $u_{n}$ and $u_{t}$ represent the overlap between the bodies at the contact point in normal and tangential directions, $v_{n}$ and $v_{t}$ are the relative velocities; $K_{n}$, $K_{t}$, $C_{n}$, $C_{t}$, are the stiffness and damping coefficients for the normal and tangential directions. The shape of $f$ depends on whether Hertzian or Hookean contact is considered; $\delta _{n}$ is the overlap in normal direction. A visual representation is reported in Fig. 2. Coulomb friction is applied as an *algebraic constraint*, namely $|F_{t}| = \mu |F_{n}|$, where $\mu $ is the friction coefficient. Hence, the equations of motion are formulated as a system of Differential Algebraic Equations (DAEs). This is a significant drawback, as the algebraic constraint places an upper limit on the time step $\Delta t$ that can be used to integrate the system. Generally, the time step must satisfy $\Delta t < \sqrt{m/k}$, where $m$ and $k$ are the characteristic mass and stiffness of the system. Problems with high stiffness may require very small time steps, leading to high computational cost. In contrast, hard-body (or non-smooth) methods treat the bodies as infinitely stiff, imposing a non-penetration condition: either the distance function $\Phi $ between the colliding bodies is zero, and the normal force $\gamma _{n}$ is greater than zero, or vice-versa. The discontinuity introduced by the contact is not treated via regularization as in the smooth model. Figure 3 provides a visual representation of the concept behind the non-smooth model. Considering the tangential contact between two bodies, one can see that for $v_{t} = 0$, the contact force is not required to assume a specific value, rather it is bounded in the range [$-F_{t,max};F_{t,max}$]: it can be referred to as a set-valued function. From a mathematical perspective, rather than differential equations, *differential inclusions* shall be considered. Hence, the problem can be formulated as a Differential Variational Inequality (DVI). Since the bodies are rigid, there are no requirements on the time step to be used in the integration. However, at each time step, a complementarity problem must be solved [6, 19], which may also lead to a significant computational burden. In hard-body methods, the dissipation resulting from the collision is captured by a single parameter, the coefficient of restitution. Although this parameter has a clear physical interpretation, it may oversimplify the problem. Specifically, the assumption of infinitely rigid bodies may not adequately represent granular materials in which cohesion forces play an important role [20]. To account for such effects while retaining the advantages of the DVI formulation, C::E implements a third model, known as the non-smooth model with compliance [20]. The contact force is still required to satisfy a convex-set inclusion, but it is related to the displacement by a classical elastic constitutive law (the extension to the elasto-plastic case is also possible [20]). Additionally, a damping matrix can be added to the model. For this reason, the input parameters are the same as in the soft-body model, i.e. friction, cohesion, stiffness and damping [20].

**Fig. 4:**
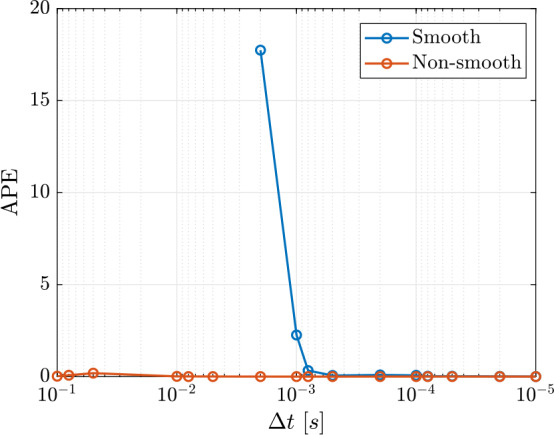
Convergence plot. The horizontal axis is in logarithmic scale. The values for the smooth method for time step $\Delta t$ larger than $2 \cdot 10^{-3}$ s are not reported because the results were non-physical. Shapes with higher resolution are used to study the convergence. (Color figure online)

In Table 2 a comparison between each model is reported, highlighting their strengths and weaknesses. The bodies in the collision scenario that will be created in the experiment are expected to be stiff, and the collision time is expected to be very short. Therefore, a hard-body method is likely to better represent the experimental outcomes. However, the flexibility offered by the non-smooth model with compliance is appealing, as it allows for the inclusion of cohesion forces, which are significant in large-scale asteroid evolution problems. The final choice is supported by a comparison of the results obtained with different models and time steps. The smooth model provides a more clear physical interpretation of the contact, but generally requires shorter time steps, resulting in longer simulation time. In [21], the following formula is given to estimate the duration of a contact event:

2 $$ \tau = 5.84 \left ( \frac{\rho (1 - \nu ^{2})}{E} \right ) ^{0.4} r \ \nu ^{-0.2} $$

where $\rho $ is the density, $\nu $ is the Poissons’ ratio, $E$ is the Young modulus and $r$ is the radius of the particles in contact. Considering values of $\rho = 2000~kg/m^{3}$, $\nu = 0.3$, $E = 100$ GPa and $r = 0.05 $ m, the contact duration can be estimated as $2.97 \cdot 10^{-4}$ s. To get accurate results with a smooth model, the time step shall be smaller than the contact duration. A convergence plot is reported in Fig. 4. The metric chosen to study the convergence is the energetic coefficient of restitution $CoR_{E}$:

3 $$ CoR_{E} = \frac{E_{k_{1}}^{+} + E_{rot_{1}}^{+} + E_{k_{2}}^{+} +E_{rot_{2}}^{+}}{E_{k_{1}}^{-} + E_{rot_{1}}^{-} + E_{k_{2}}^{-} + E_{rot_{2}}^{-}} $$

where $E_{k_{i}}^{-}$, $E_{rot_{i}}^{-}$, $E_{k_{i}}^{+}$ and $E_{rot_{i}}^{+}$ are the translational kinetic and rotational energy pre- and post-collision for each body. The necessity of such definition is discussed at the end of the current Section. The Absolute Percentage Error (APE) is shown, computed as $APE = |(CoR_{E, \Delta t} - CoR_{E, ref}) / CoR_{E, ref}| \cdot 100\%$, where $CoR_{E, \Delta t}$ is the $CoR_{E}$ as function of the time step and $CoR_{E, ref}$ is the value obtained for the shortest time step considered, namely $10^{-5}$ s. It is important to stress that the *APE* refers to convergence in this case, not accuracy. It can be noticed that for the smooth model convergence is achieved for time steps shorter than $10^{-4}$ s, in agreement with the result from Eq. (3); in this case the wall-clock time is 29.81 s. On the other hand, there is not an evident dependency on the time step for the non-smooth model. Considering a time step $\Delta t = 10^{-3}$ s, the wall-clock time is 5.65 s. Since the purpose of the project is to calibrate the contact models to reproduce the experiments, optimization algorithms will be used, that require to run a large number of simulations. The shorter simulation time required by the non-smooth model makes it more suitable for future applications, hence it has been used also to generate the benchmark scenario. When compared to the non-smooth model with zero compliance, no significant difference has been observed for the scenario presented. Nevertheless, compliance is added because it is considered a promising approach to combine the advantages of both smooth and non-smooth methods. However, each contact model will be tested against experimental data to further evaluate their respective advantages and limitations.

**Table 2:** Contact models comparison

Model	Pros	Cons	Reference
Smooth	Standard DAEs integrators can be used Direct physical interpretation of contact parameters	Short time steps required for problems with high stiffness Complex formulas to tune contact parameters	[9, 19]
Non-smooth	Time step not dependent on the problem sizeLarge problems can be handled	DVI solvers requiredPoor physical interpretation of contact parameters	[10, 19]
Non-smooth with compliance	Retains advantages of DVI formulation while enhancing physical representation	Low maturity	[20]

Some of the parameters used are reported in Table 3. For all other parameters, default values have been used. Note that the $CoR_{input}$ required as input by C::E material properties refers to the ratio between the after-contact velocity, $v_{N}^{+}$, and before-contact, $v_{N}^{-}$, along the impact direction:

4 $$ CoR_{input} = \frac{v_{N}^{+}}{v_{N}^{-}} $$

**Table 3:** Simulation parameters for the benchmark model of the digital twin

$CoR_{input}$ [-]	Static Friction [-]	Compliance [m/N]	Time [s]
0.6	0.8	1e-4	2.5

**Fig. 5:**
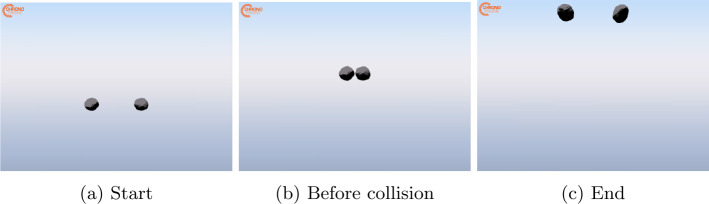
Snapshots of the simulation at different times

**Fig. 6:**
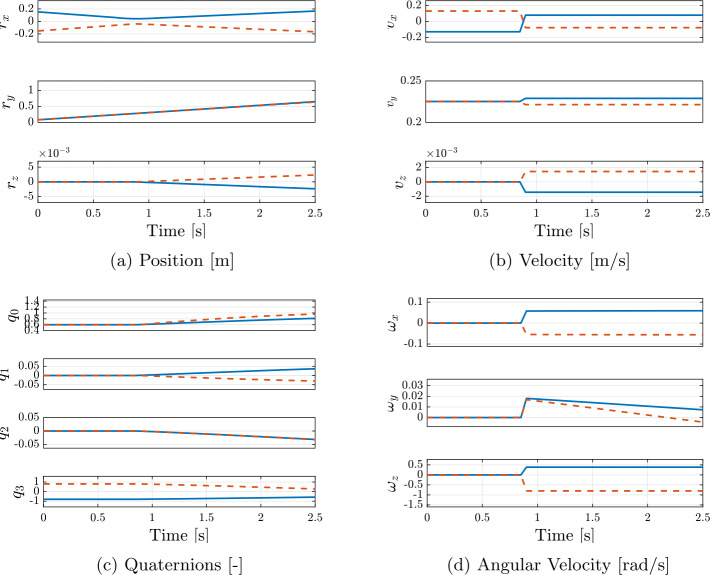
Evolution of the states of the simulated benchmark model, for body 1 (solid blue line) and body 2 (dashed red line). Scalar-first convention for the quaternions is used. (Color figure online)

**Fig. 7:**
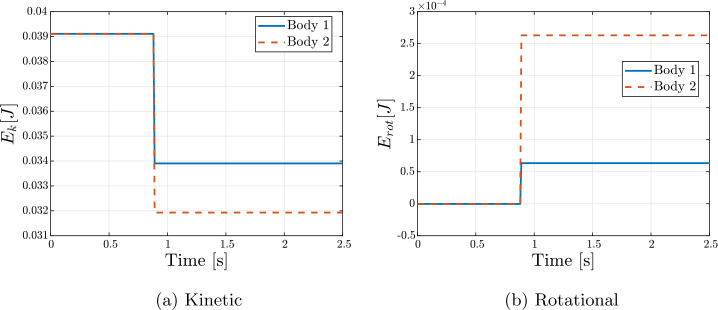
Kinetic and Rotational Energy components of the simulated benchmark model

The nominal initial conditions are reported in Table 4. Snapshots from the simulation are shown in Fig. 5. The evolution of the cobbles’ states for the nominal case is reported in Fig. 6. It can be seen that the collision introduces a discontinuity in the evolution of all states. This is reflected in the trend of kinetic and rotational energy, shown in Fig. 7. The decrement in the kinetic component is counteracted by a gain in the rotational component. A definition of the CoR that solely considers the velocity in the direction normal to the impact is inadequate to characterize this dissipation. For this reason, $CoR_{E}$ has been introduced in Eq. (3), which accounts for both the dissipation and the conversion of kinetic energy into rotational energy. This parameter is particularly important for investigating contact dynamics in the asteroid scenario, as it is one of the main observables during the interaction of a lander and the asteroid surface. In addition, the estimation of the CoR allows one to retrieve other surface mechanical properties, such as soil strength and cohesion [22, 23]. A simple assessment of the physical reliability of the model has been performed by studying the results of the simulation obtained varying the relative inclination of the bodies and the input $CoR_{input}$. Inclination angles in the range $\boldsymbol{\alpha} \in [0^{\circ}, 15^{\circ}, 30^{\circ}, 60^{\circ}]$ and normal coefficient of restitution in $CoR_{input} \in [0.6, 0.7, 0.8, 0.9]$ have been considered. The value of $CoR_{E}$ for each combination is reported in Table 5. As expected, the $CoR_{E}$ increases with increasing inclination. Specifically, the velocity component along the $x$-direction decreases for larger values of $\alpha $, leading to less dissipation during the collision. Similarly, higher values of $CoR_{input}$ result in higher $CoR_{E}$. Additionally, Table 6 presents the ratio between $v_{N}^{+}$ and $v_{N}^{-}$, computed a posteriori from the simulation outcomes. The obtained values are in good agreement with the input values. Any small deviations can be attributed to the fact that contact occurs at multiple points, while the input value for C::E’s $CoR_{input}$ is defined for a single contact point. These results confirm that the model is consistent with the expected physical behavior of the system.

**Table 4:** Initial conditions for the cobbles’ states in the benchmark model

	$\boldsymbol{r}_{0}$ [m]	$|| \boldsymbol{v}_{0} ||$ [m/s]	*α* [deg]	$\boldsymbol{q}_{0}$ [-]	$\boldsymbol{\omega}_{0}$ [rad/s]
Body 1	$[-0.153, 0.08, 0]^{\top}$	0.26	60	$[0.6, 0, 0, -0.8]^{\top}$	$[0, 0, 0]^{\top}$
Body 2	$[0.153, 0.08, 0]^{\top}$	0.26	60	$[0.6, 0, 0, 0.8]^{\top}$	$[0, 0, 0]^{\top}$

**Table 5:** $CoR_{E}$ for different values of $\alpha $ and $CoR_{input}$

	*α* = 0^∘^	*α* = 15^∘^	*α* = 30^∘^	*α* = 60^∘^
$CoR_{input} = 0.6$	0.3972	0.3956	0.5376	0.8458
$CoR_{input} = 0.7$	0.5256	0.5154	0.6525	0.8842
$CoR_{input} = 0.8$	0.6812	0.6535	0.7772	0.9257
$CoR_{input} = 0.9$	0.8843	0.8107	0.8897	0.9632

**Table 6:** Ratio between $v_{N}^{+}$ and $v_{N}^{-}$ recovered from simulation outcomes, for different values of input $CoR_{input}$ and $\alpha $

	*α* = 0^∘^	*α* = 15^∘^	*α* = 30^∘^	*α* = 60^∘^
$CoR_{input} = 0.6$	0.6158	0.5643	0.6052	0.6047
$CoR_{input} = 0.7$	0.7098	0.6619	0.7170	0.7166
$CoR_{input} = 0.8$	0.8096	0.7592	0.8227	0.8224
$CoR_{input} = 0.9$	0.9250	0.8571	0.9096	0.9091

## Approach and methodology

**Fig. 8:**
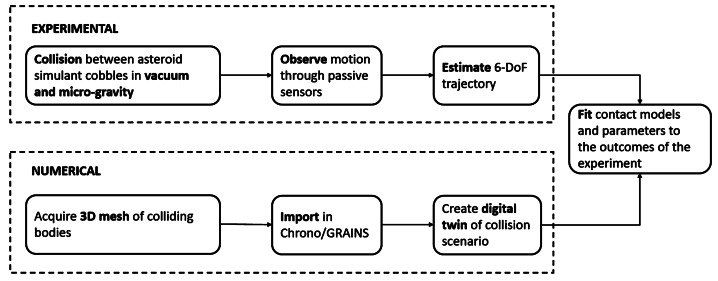
Layout of the validation campaign, encompassing both experimental and numerical activities

This Section outlines and discusses the requirements for each component of the experiment. It includes a detailed description of the facility, the release mechanism necessary to induce a collision, and the sensors required to monitor the motion. Additionally, the approach used to estimate the trajectory of the cobbles is presented. The layout of the activities planned for the experimental validation campaign is shown in Fig. 8.

### Facility

The driving criterion for the choice of the facility is related to the micro-gravity quality achievable. Indeed, the typical gravitational acceleration experienced by particles on asteroids with size > 100 m is in the order of $10^{-6} - 10^{-5} g_{0} $ [24]. The primary criterion for selecting the facility is the quality of microgravity it can provide. The typical gravitational acceleration experienced by particles on asteroids larger than 100 m is on the order of $10^{-6} - 10^{-5} g_{0}$ [24]. This requirement can be met by the ZARM Drop Tower in Bremen [25, 26], where several successful asteroid science experiments have been conducted, demonstrating the facility’s reliability for such studies [27, 28].

The experiment is housed within a capsule, which is equipped with sensors and a data acquisition system. The capsule is pressurized to standard atmospheric pressure, while the air is evacuated from the tower before the drop to minimize air drag and achieve the aforementioned levels of micro-gravity. In the standard drop mode, the capsule is elevated to the top of the tower and released in a 110-meter drop, creating microgravity conditions for approximately 4.7 seconds, corresponding to the duration of free fall.

To replicate the asteroid environment with high fidelity, the experiment must also be conducted in vacuum conditions. Since the drop capsule is pressurized, the experiment needs to be placed inside a vacuum chamber, which is then mounted within the drop capsule. The vacuum chamber that was previously employed in microgravity tests for the Hayabusa II mission landers will be borrowed from the Japan Aerospace Exploration Agency (JAXA) for this experiment.

### Release mechanism

**Fig. 9:**
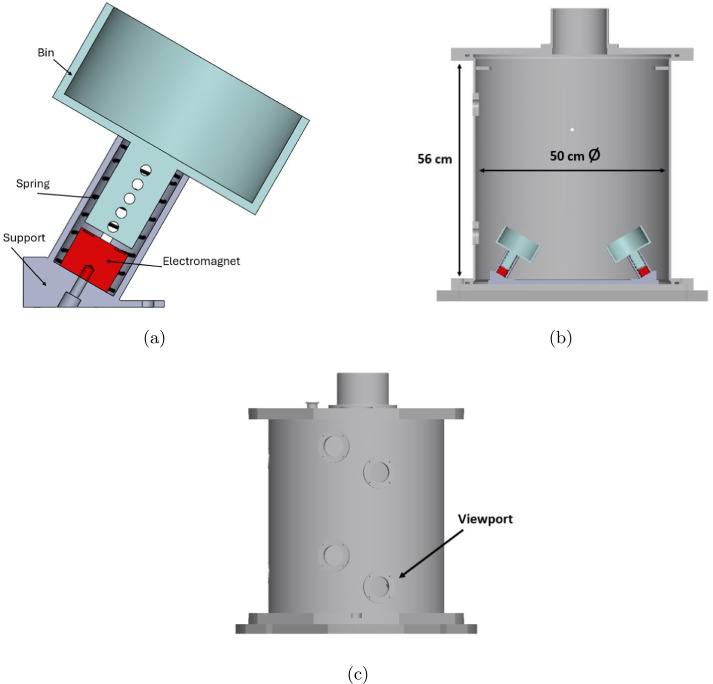
CAD model sectional view of release mechanism (a) and system integrated in JAXA vacuum chamber (b). CAD model of JAXA vacuum chamber with view-ports highlighted (c)

A release mechanism is necessary to push the cobbles against each other and obtain a collision. Following the approach used in previous experiments at ZARM [27], the initial energy will be provided by two compression springs. A sectional view of the CAD model of the release mechanism is reported in Fig. 9. Each spring will be mounted inside an external cylindrical guide along intersecting paths. The cobbles will be housed in bins that slide within the cylindrical guide. Each bin is equipped with a guiding rod featuring through-holes at regular intervals. A pin will be inserted into these holes to compress the spring and lock the mechanism. The release velocity can be adjusted by inserting the pin into different holes. The clamping force needed to keep the spring compressed before release will be provided by an electromagnet (EM) attached to the bottom of the cylindrical guide. Prior to release, the EM is connected to a power source, and current flows through the EM’s coil, generating a magnetic force normal to the EM surface, which compresses the spring. After the capsule is released from the top of the tower, there is a brief transient period (approximately 0.5 s), during which the acceleration experienced by the capsule decreases from 1 to $10^{-6}$ $g_{0}$. Once this transient phase has passed, the current is cut off, allowing the spring to release and push the cobble-bin system. Upper and lower bounds for the release velocity have been established to ensure compatibility with the internal dimensions of the vacuum chamber and the duration of the drop. Given the aforementioned transient period, approximately 4 seconds are available to observe the cobbles’ motion. Hence, a maximum duration of 2 s has been set on both the pre and post impact trajectories. With reference to the initial conditions in Table 4, these constraints lead to a minimum release velocity of 15 cm/s. The upper bound for the release velocity has been set at 30 cm/s to ensure sufficient observation time during the initial trajectory arc and to facilitate the tracking procedure. Further details are provided in Sect. 3.3. The characteristics of the spring necessary to obtain a release velocity in this range have been estimated by considering the balance between kinetic and elastic energy. For a body with a mass of 1 kg, the desired release velocity range can be achieved with a spring constant of approximately 0.2 N/mm and a pre-load between 1 and 2 cm. The exact characteristics of the spring and the optimal pre-load will be determined through laboratory tests. It is important to note that the force required to push the cobbles during ground testing is greater than that required in microgravity conditions. Therefore, two different sets of springs are being tested: one for the in-lab tests and another for the drop campaign. Additionally, since each cobble is a unique specimen with a slightly different mass, a small variation in release velocity is expected for each cobble. This variation is not problematic, as long as the velocities remain within the specified bounds.

### Sensors

#### Cameras

The choice of cameras as sensors is based on the need to minimize interaction with the colliding bodies. Markers will be opportunely placed on the cobbles and tracked to obtain measurements for full-state trajectory reconstruction. High-resolution and high-speed cameras are necessary to achieve optimal tracking performance. An additional critical requirement is that the cameras must withstand the $50~g_{0}$ deceleration experienced by the capsule at the bottom of the drop tower [25]. The ZARM facility offers Phantom Miro C321 cameras as standard equipment. However, experimenters are only permitted to place these cameras outside the vacuum chamber (see Fig. 9). Therefore, GoPro cameras will be used inside the vacuum chamber to provide additional visual coverage. GoPro cameras offer the best balance between cost and performance for high-g applications, with a maximum frame rate of 240 fps. This frame rate justifies the upper bound for the release velocity, as discussed in Sect. 3.2. Indeed, most tracking algorithm, e.g. Kanade-Lucas-Tomasi [29], perform at best when the motion between successive frames is minimal. For a velocity of 30 cm/s and a frame rate of 240 fps, the displacement between frames is approximately 1.25 mm, which is deemed sufficient for good tracking performance. The Phantom Camera Control (PCC) Software will be used to control, calibrate and synchronize the cameras. Moreover, it can be exploited to perform image processing and to track the markers on the cobbles. Key specifications of the cameras are reported in Table 7. Camera calibration is necessary to estimate intrinsic and extrinsic camera parameters [30]. Extrinsic parameters relate the world coordinate system to the camera reference frame. These parameters are typically represented by a rotation matrix $\boldsymbol{R}_{CN}$, which describes the rotation of the camera frame with respect to the inertial frame, and a translation vector $\boldsymbol{t}_{CN}$, which defines the displacement from the origin of the inertial frame to the origin of the camera frame. On the other hand, intrinsic parameters are required to transform the position of an object from the 3D camera reference frame to the 2D image plane. These include the focal length along horizontal and vertical axes $f_{x}$ and $f_{y}$, the sensor dimensions, $s_{x}$ and $s_{y}$, and the displacement of the origin of the image coordinate system relative to the center of the camera reference frame, $c_{x}$ and $c_{y}$. The skew parameter $s$ is usually assumed to be null [30]. A detailed explanation about how the camera parameters appear in the measurement model will be given in Sect. 3.5. The Matlab camera calibration application, whose algorithms are based on the approach presented in [31], is used to obtain the intrinsic and extrinsic parameters.

**Table 7:** Camera Data

Model	Max frame rate [fps]	Resolution [px]	FoV [deg]
Phantom Miro C321	1480	1920 × 1080	N/A
GoPro Hero 12	240	2704 × 1520	87 × 54

#### 3D scanner

**Fig. 10:**
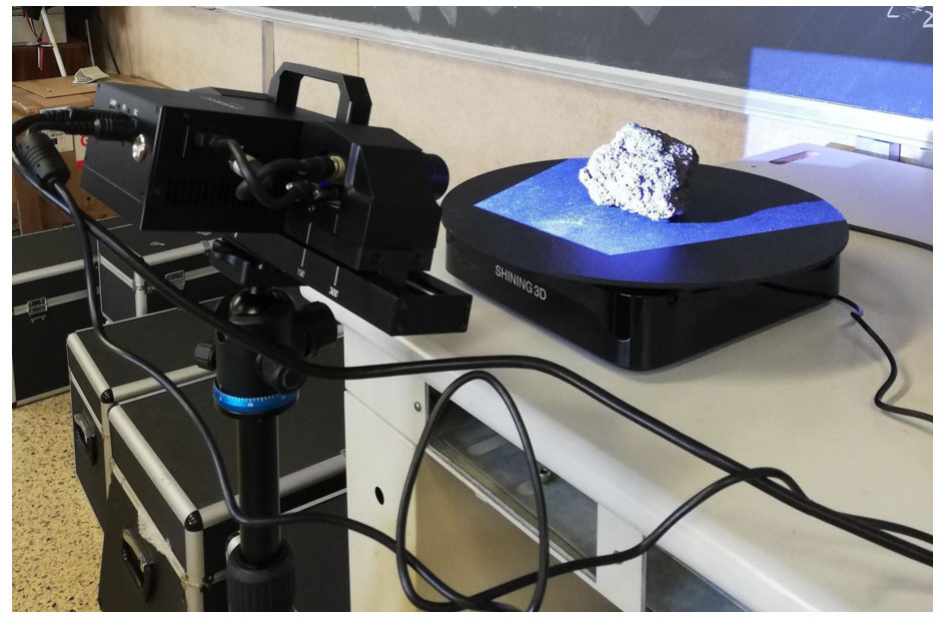
3D scanner during operations

A 3D scanner will be used to acquire the shape of the simulant objects, with the resulting data provided to the N-body code in .obj file format. The selected sensor is a structured-light scanner with a declared single shot accuracy of 0.035 mm. A LED projector projects light patterns onto the object to be scanned. The light is deformed by the natural texture of the object. The two cameras observe how the light pattern is deformed and, upon calibration, are able to triangulate the points and accurately reconstruct their 3D position. Once the point cloud is acquired, it is processed to obtain a watertight mesh. In Fig. 10 the scanner in operation is shown. Acquiring the shape is essential not only for simulating contact dynamics, but also for the estimation technique described in Sect. 3.5. Specifically, the state estimation algorithm requires precise knowledge of the markers’ positions within the body frame, as well as the inertia properties of each cobble. All of this information will be obtained using the 3D scanner or deduced from scanner measurements, under the assumption of uniform density.

### Simulant material

To simulate asteroid material accurately, it is essential to use irregular cobbles with sharp edges. Indeed, due to the absence of an atmosphere, rocks on asteroid are unlikely to undergo weathering. Concerning the dimensions of the colliding cobbles, they shall be compliant with the internal dimensions of the vacuum chamber. However, an excessively small size would make the observation through cameras challenging. A good compromise is achieved with a characteristic length in the order of 8-15 cm. A further requirement is that the chemical composition of the simulants shall resemble that of asteroid material. The asteroid simulant material provided by Space Resource Technologies (previously Exolith Lab) meets these requirements. The chemical composition of their simulants is designed to mimic the composition of carbonaceous chondrites found in known meteorites. The CI-E simulant is based on the Orgueil meteorite composition [32], while the CM-E simulant is based on the Murchison meteorite [32, 33]. These simulants are prepared by crushing and drying raw material until cobbles of the desired size are obtained, following the procedure outlined in [34]. Hence, a set of cobbles has been purchased from this supplier.

However, for in-lab testing, a cost-effective alternative is necessary. Therefore, a second set of cobbles has been sourced from Mount Etna. These rocks, which come from recent volcanic eruptions, have been exposed to weathering only in recent times. Although their chemical composition differs from that of meteorite samples, their mechanical properties are expected to closely resemble those of asteroid material.

### Estimation algorithm

The objective is to estimate the full 6-dof trajectory of the cobbles both before and after the collision. The discontinuity introduced by the collision makes it challenging to estimate the entire trajectory in one step. Therefore, it is beneficial to split the estimation into two separate ballistic phases: before and after the collision. During this process, the energy across the impact will be used to estimate the coefficient of restitution. This approach allows for the use of any software for trajectory estimation, as the collision modeling itself is not required. Once the trajectory is estimated, the digital twin created in C::E will be calibrated to accurately reproduce the observed trajectory.

#### Measurement model

**Fig. 11:**
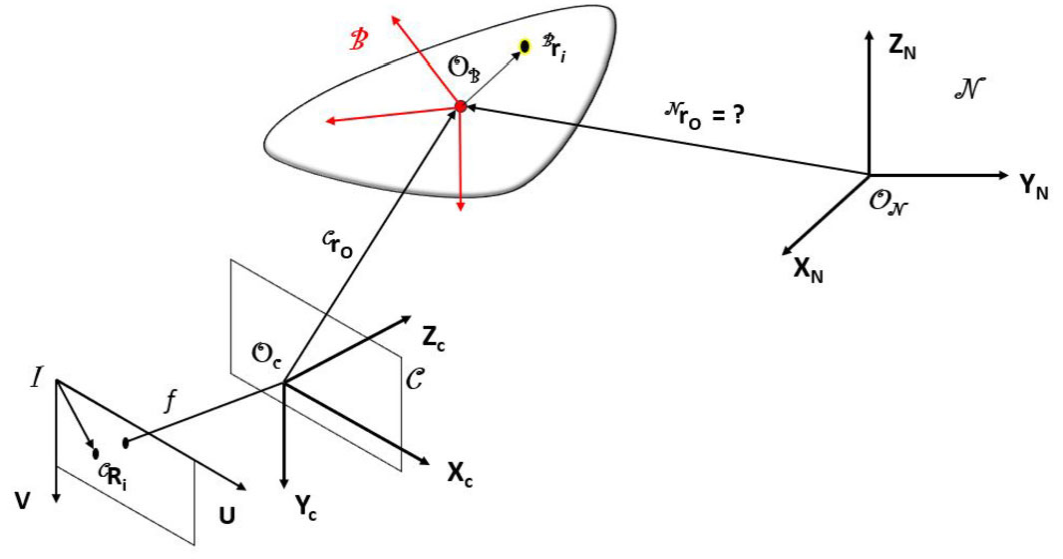
Reference frames. *N* is the inertial reference frame, *B* is the body reference frame, *C* is the camera ref. frame and *I* is the image plane. Note that this picture is just intended as a general description; the relative position of each reference frame is not representative of the real experiment set up

The estimation strategy chosen is inspired by vision-based navigation techniques [35–37]. As mentioned in Sect. 3.3, the position of each marker in the body frame of the cobbles is acquired by the scanner, as well as the inertia properties. Each marker will be tracked through the PCC Software tracking algorithm. The algorithm is based on correlation. In the first image, a region of interest centered on the chosen point is selected. Then, the algorithm is able to correlate the same region in subsequent frames automatically, although supervision is always recommended to avoid excessive drifting. The quality of automatic tracking depends on the ratio between the velocity of the motion and the frame rate of the cameras. This is the reason why high-speed cameras are considered, as discussed in Sect. 3.3. The available measurements are the 2D pixel coordinates of each marker in the camera plane ${^{\textit{I}}\boldsymbol{R}_{i}^{*}} $. In order to build the residual vector, it is needed to convert the states $\boldsymbol{x}$ into pixel coordinates. This requires a series of transformations between the reference frames represented in Fig. 11. The position of each marker in the body frame is converted into the inertial frame via the equation:

5 $$ {^{\textit{N}}}\boldsymbol{r}_{i} = {^{\textit{N}}}\boldsymbol{r}_{CoM} + \boldsymbol{A}_{NB} {^{ \textit{B}}}\boldsymbol{r}_{i} $$

where ${^{\textit{N}}}\boldsymbol{r}_{CoM}$ is the position of the cobble’s center of mass (CoM) expressed in inertial frame, ${^{\textit{N}}}\boldsymbol{r}_{i}$ and ${^{\textit{B}}}\boldsymbol{r}_{i}$ are the position of the i-th marker respectively in the inertial and body frame, and $\boldsymbol{A}_{NB}$ represents the attitude from the body frame to the inertial frame. It is then necessary to project the position of the marker in the reference frame of the camera:

6 $$ {^{\textit{C}}}\boldsymbol{r}_{i} = {^{\textit{C}}}\boldsymbol{t}_{CN} + \boldsymbol{R}_{CN} {^{ \textit{N}}}\boldsymbol{r}_{i} $$

where matrix $\boldsymbol{R}_{CN}$ and vector ${^{\textit{C}}}\boldsymbol{t}_{CN}$ represent the roto-translation applied to express a point from the inertial reference frame to the camera reference frame, ${^{\textit{C}}}\boldsymbol{r}_{i} = [x_{c}, y_{c}, z_{c}]^{\top}$ is the position of the i-th marker in the camera reference frame. Note that $\boldsymbol{R}_{CN}$ and ${^{\textit{C}}}\boldsymbol{t}_{CN}$ are the extrinsic parameters introduced in Sect. 3.3. At this stage, simple rigid transformations have been applied to transform the position of each marker in body frame, provided by the scanner, into the position in the camera reference frame. It is now necessary to project the 3D position in camera reference frame onto the 2D image plane to recover the position in pixel coordinates. The first step requires to divide the $x$ and $y$ components by the distance from the camera frame:

7 $$\begin{aligned} U_{i}^{u} = \frac{x_{c}}{z_{c}} \\ V_{i}^{u} = \frac{y_{c}}{z_{c}} \end{aligned}$$

where $U_{i}^{u}$ and $V_{i}^{u}$ are the normalized coordinates in camera reference frame. It is then necessary to account for the distortion introduced by the sensor, according to the radial distortion model in [38]:

8 $$\begin{aligned} U_{i}^{d} = U_{i}^{u} (1 + w_{1} \rho _{i}^{2} + w_{2} \rho _{i}^{4} + w_{3} \rho _{i}^{6}) \\ V_{i}^{d} = V_{i}^{u} (1 + w_{1} \rho _{i}^{2} + w_{2} \rho _{i}^{4} + w_{3} \rho _{i}^{6}) \end{aligned}$$

where $\rho _{i} = \sqrt{(U_{i}^{u})^{2} + (V_{i}^{u})^{2}}$ and $w_{1}$, $w_{2}$, $w_{3}$ are distortion parameters recovered through camera calibration. $U_{i}^{d}$ and $V_{i}^{d}$ are the coordinates in camera frame, normalized by the distance and corrected for the distortion. The projection in the image plane can be obtained applying the following equations:

9 $$\begin{aligned} \boldsymbol{R}_{i} = \begin{pmatrix} f_{x} \cdot U_{i}^{d} + c_{x} \\ f_{y} \cdot V_{i}^{d} + c_{y} \end{pmatrix} \end{aligned}$$

where $f_{x}$, $f_{y}$, $c_{x}$ and $c_{y}$ are the intrinsic camera parameters introduced in Sect. 3.3 and $\boldsymbol{R}_{i}$ is the vector embedding the pixel coordinates in horizontal and vertical direction.

#### Estimator

A batch estimation technique can be applied, as the estimator is not required to run in real-time [39]. The trajectory is numerically propagated from initial to final time over each ballistic arc, given the initial conditions $\boldsymbol{x}_{0} = [\boldsymbol{r}^{0}_{CoM}, \boldsymbol{v}^{0}_{CoM}, \boldsymbol{p}^{0}_{NB}, {^{ \textit{B}}}\boldsymbol{\omega}^{0}_{NB}]^{\top}$. Note that the Modified Rodrigues Parameters $\boldsymbol{p}^{0}_{NB}$ are used to parametrize the attitude, rather than quaternions. This is done to avoid introducing the unitary norm constraint, following the approach exposed in [37]. The position of the tracked vertices is then converted into the image plane using the measurement model outlined in the previous paragraph, to obtain $\boldsymbol{R}_{ij}$, the pixel coordinates of marker $i$ recovered from camera $j$. Given the measurements ${^{\textit{I}}\boldsymbol{R}_{ij}^{*}} $, the array of the residuals at time $t_{k}$ is built as:

10 $$ \boldsymbol{J}_{t_{k}} = \begin{Bmatrix} \boldsymbol{R}_{11} - {^{\textit{I}}\boldsymbol{R}_{11}^{*}} \\ \vdots \\ \boldsymbol{R}_{nm} - {^{\textit{I}}\boldsymbol{R}_{nm}^{*}} \end{Bmatrix} $$

Eventually, the residual array at each time can be collected in the global residual array:

11 $$ \boldsymbol{J}_{tot} = \begin{Bmatrix} \boldsymbol{J}_{t_{1}} \\ \vdots \\ \boldsymbol{J}_{t_{N}} \end{Bmatrix} $$

At this stage, the initial state estimation problem can be stated as:

12 $$ \begin{aligned} \min _{\boldsymbol{x}_{0}} \quad & \boldsymbol{J}_{tot}^{\top}\boldsymbol{J}_{tot} \end{aligned} $$

and the solution can be recovered applying standard least-squares techniques [39]. To solve this problem, derivatives are necessary which can be numerically computed or analytically derived as proposed in [37].

## Results from numerical tests

The purpose of this Section is to present and validate the strategy implemented to estimate the trajectory and coefficient of restitution. The goal is to apply the same procedure to the experimental data to evaluate the accuracy of the estimation, as further discussed in Sect. 4.3.

### Trajectory estimation

The estimation algorithm is tested through numerical simulations. The nominal trajectory of the collision scenario presented in Sect. 2 is used as a reference trajectory for the estimation. A subset of the 1348 vertices that make up the bodies’ mesh is selected to generate the measurements, simulating the positions of the markers that will be used in the actual experiment. To account for potential occlusions of the tracked vertices, a simple model has been implemented. Specifically, the $i$-th marker is considered occluded when:

13 $$ \beta _{ij} = \arccos \Big( \frac{{^{\textit{C}}}\boldsymbol{r}_{ij} \cdot {^{\textit{C}}}\boldsymbol{r}_{CoM}}{||{^{\textit{C}}}\boldsymbol{r}_{ij} \cdot {^{\textit{C}}}\boldsymbol{r}_{CoM}||} \Big) < 90^{\circ} $$

i.e., if the angle between the vectors from the center of the $j$-th camera reference frame to the CoM, ${^{\textit{C}}}\boldsymbol{r}_{CoM}$, and to the $i$-th marker, ${^{\textit{C}}}\boldsymbol{r}_{ij}$, is lower than $90^{\circ}$. All cameras have been modeled with the same resolution as Phantom Miro C321 cameras; however, since no data are available, the FoV of GoPro cameras has been used (see Table 7).

The time interval is divided into two arcs, whose bounds are reported in Table 8. A time frame of $0.4~s$ is left out of the estimation, in order to avoid the sections of the trajectory right before and after the collision. The measurements are obtained by applying the measurement model presented in Sect. 3.5 to the reference trajectory. Lighting conditions have not been simulated. Indeed, the experiment is going to be performed with optimal lighting conditions controlled by the experimenters. These conditions will be tested during Earth-gravity tests performed in our premises before the actual experimental campaign. Noise is introduced to account for two sources of uncertainty:

- **Markers’ positions**: these positions will be acquired from the 3D model of each cobble, which introduces errors related to both the scanning process and the manual placement of the markers. Then: ${^{\textit{B}}}\boldsymbol{r}_{i} \sim \mathcal{N}({^{\textit{B}}}\bar{\boldsymbol{r}}_{i}, \sigma _{V})$. A value of $\sigma _{V} = 0.67$ mm is assumed for all scenarios. Taking into account a reference value of 2 mm for the markers’ size, this value ensures that the uncertainty is limited to the size of the marker ($3 \sigma _{V} \simeq 2$ mm).
- **Tracking**: After the markers’ trajectories are projected into the frame of each camera, additional noise is introduced to account for the tracking error that is expected in the real experiment. This is modeled as: $\boldsymbol{R}_{i} \sim \mathcal{N}(\bar{\boldsymbol{R}}_{i}, \sigma _{px})$, where $\sigma _{px}$ represents the tracking noise, assumed to be the same for all cameras. Results are presented for varying levels of $\sigma _{px}$.

In total, the measurements have been generated at 14 instants on the first arc and at 30 instants on the second one, meaning that they have been sampled at a significantly lower frequency with respect to the capability of the real cameras. This is done to test the performance of the estimation procedure in a challenging condition. The initial guess is given by sampling from a uniform distribution centered in the reference initial state. The bounds are listed in Table 9. The algorithm has been tested under the following scenarios:

- **Scenario 1**: cameras A and B in the lower view-ports, camera C in the upper-left view-port; 7 vertices tracked.
- **Scenario 2**: camera A in lower-right viewport, camera C in upper-left viewport of the JAXA vacuum chamber (see Fig. 9), camera B on the opposite side of the vacuum chamber; 7 vertices tracked.
- **Scenario 3**: cameras A and B in the lower and upper left viewport; 7 vertices tracked. In this scenario a challenging condition has been tested, in which only two cameras are available.

**Fig. 12:**
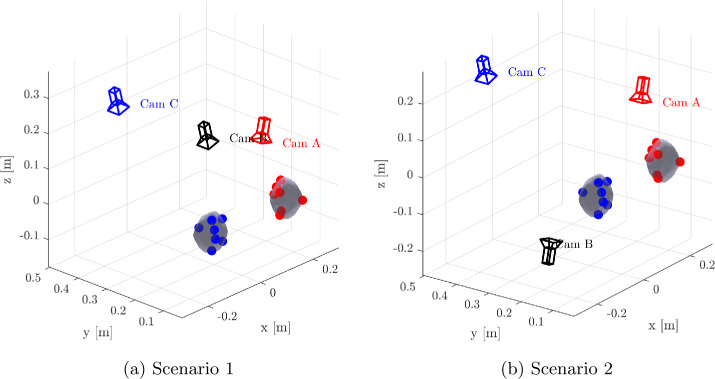
Camera placement scenarios. Red and blue dots represent the tracked vertices on body 1 and 2 respectively. (Color figure online)

**Fig. 13:**
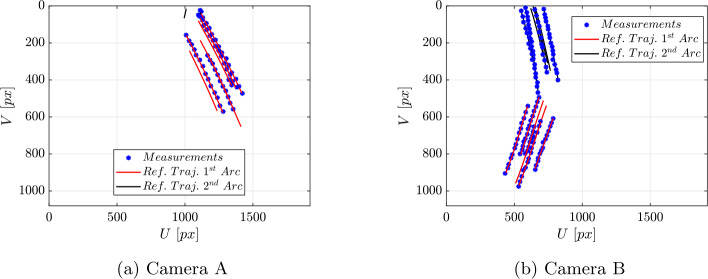
Reference trajectories of the tracked vertices of body 1 projected in image plane and measurements obtained introducing noise, scenario 2, $\sigma _{px} = 3$ px. (Color figure online)

**Fig. 14:**
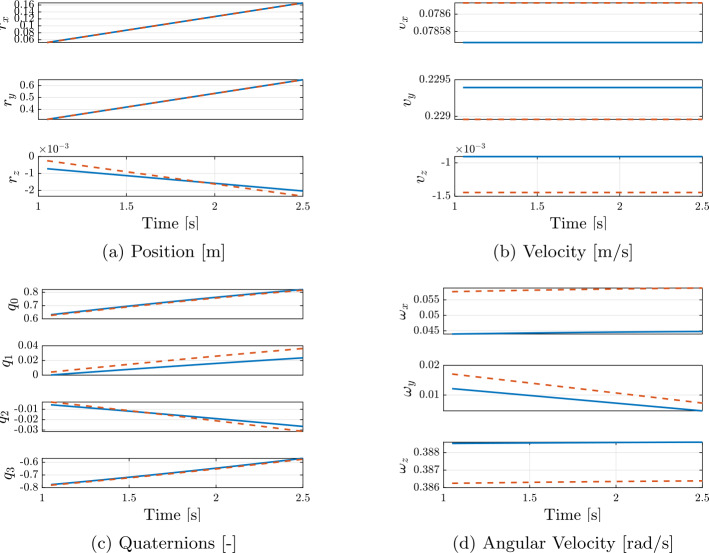
Estimated (solid blue line) vs True (dashed red line) State evolution on Second Arc, Body 1. Relative to scenario 2, with $\sigma _{px} =$ 3 px. (Color figure online).

A representation of camera geometry in scenario 1 and 2 is shown in Fig. 12. An example of the vertices position projected in the image plane is presented in Fig. 13. It is worth noting that in Camera A, only the vertices’ trajectory from the first arc is visible. This is due to the fact that, after the collision, the body moves out of Camera A’s field of view. As a result, Camera A can only provide information about the pre-collision ballistic trajectory. For each of these scenarios, different noise levels have been applied to the measurements. The root mean square (rms) of the difference between the states propagated from the estimated initial state and the true values is used as the error metric. Results for position and angular velocity estimation for each case are reported in Table 10, Table 11, and Table 12. The trend of the estimated states relative to the true ones is shown in Fig. 14, for scenario 2 with $\sigma _{px} = 3 $ px. The rms error is generally lower in the case with $\sigma _{px} = 1$ px, while no significant differences can be observed between the cases with 2 and 3 px. As expected, the rms error is generally higher in scenario 3. However, given the dimensions of the problem, the estimation can be regarded as successful in all the scenarios tested. These findings provide valuable insights into the optimal placement of cameras for the actual experiment. In the next section, a statistical analysis of the results obtained is presented.

**Table 8:** Time intervals

First arc		Second arc	
$t_{0}^{1st}$ [s]	$t_{f}^{1st}$ [s]	$t_{0}^{2nd}$ [s]	$t_{f}^{2nd}$ [s]
0	0.65	1.05	2.5

**Table 9:** Deviations around initial conditions used to compute the bound for sampling the initial guess

	$\Delta \boldsymbol{r}~\mathrm{[m]}$	$\Delta \boldsymbol{v}~\mathrm{[m/s]}$	$\Delta \boldsymbol{p}~\mathrm{[-]}$	$\Delta \boldsymbol{\omega}~\mathrm{[rad/s]}$
First arc	$\pm [0.1, 0.1, 0.1]^{\top}$	$\pm [0.1, 0.1, 0.1]^{\top}$	$\pm [0.1, 0.1, 0.1]^{\top}$	$\pm [0.1, 0.1, 0.1]^{\top}$
Second arc	$\pm [0.1, 0.1, 0.1]^{\top}$	$\pm [0.1, 0.1, 0.1]^{\top}$	$\pm [0.1, 0.1, 0.1]^{\top}$	$\pm [0.1, 0.1, 0.5]^{\top}$

**Table 10:** Root Mean Square error norm - Scenario 1. Position in [m], angular velocity in [rad/s]

		$\sigma _{px} = 1$ px		$\sigma _{px} = 2$ px		$\sigma _{px} = 3$ px	
		||***r***|| (10^−4^)	||***ω***||	||***r***|| (10^−4^)	||***ω***||	||***r***|| (10^−4^)	||***ω***||
First Arc	Body 1	6.0596	0.0473	5.4501	0.0470	6.9741	0.0556
	Body 2	7.8178	0.0112	5.6320	0.0459	19.551	0.0309
Second Arc	Body 1	7.8990	0.0190	9.5734	0.0138	6.9574	0.0114
	Body 2	5.0795	0.0127	15.153	0.0751	11.985	0.0571

**Table 11:** Root Mean Square error norm - Scenario 2. Position in [m], angular velocity in [rad/s]

		$\sigma _{px} = 1$ px		$\sigma _{px} = 2$ px		$\sigma _{px} = 3$ px	
		||***r***|| (10^−4^)	||***ω***||	||***r***|| (10^−4^)	||***ω***||	||***r***|| (10^−4^)	||***ω***||
First Arc	Body 1	6.0596	0.0473	5.4501	0.0470	6.9741	0.0556
	Body 2	7.8178	0.0112	5.6320	0.0459	19.551	0.0309
Second Arc	Body 1	7.8990	0.0190	9.5734	0.0138	6.9574	0.0114
	Body 2	5.0795	0.0127	15.153	0.0751	11.985	0.0571

**Table 12:** Root Mean Square error norm - Scenario 3. Position in [m], angular velocity in [rad/s]

		$\sigma _{px} = 1$ px		$\sigma _{px} = 2$ px		$\sigma _{px} = 3$ px	
		||***r***|| (10^−4^)	||***ω***||	||***r***|| (10^−4^)	||***ω***||	||***r***|| (10^−4^)	||***ω***||
First Arc	Body 1	5.5584	0.0206	17.164	0.0682	10.826	0.0359
	Body 2	23.172	0.1178	12.521	0.0760	16.706	0.0593
Second Arc	Body 1	10.470	0.0282	12.211	0.0174	7.1583	0.0271
	Body 2	13.222	0.0392	12.703	0.0365	12.497	0.0538

### Coefficient of restitution estimation - statistical analysis

**Fig. 15:**
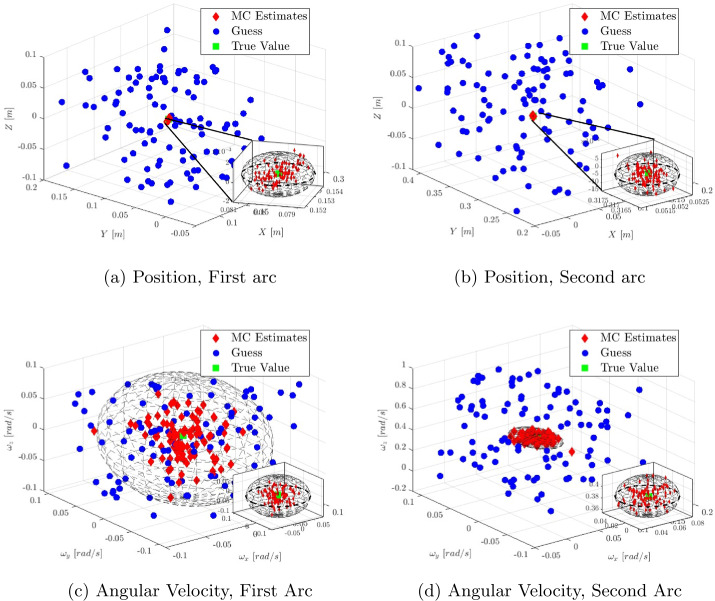
Estimated states and $\pm 3 \sigma $ ellipsoids obtained with 100 samples in scenario 2, with $\sigma _{px} =$ 3 px. Angular velocities in body frame

**Fig. 16:**
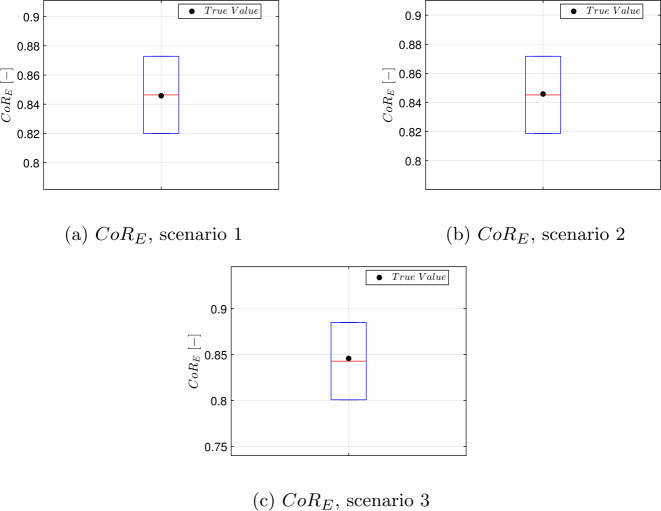
Statistical analysis on $CoR_{E}$ estimate, with $\sigma _{px} = 3$ px in all scenarios. The box corresponds to the $\pm 3 \sigma $ confidence interval

Since the primary objective is to estimate the contact dynamics of the experiment, the uncertainty in the estimation of the energetic coefficient of restitution has been assessed through Monte Carlo (MC) analysis on the previously presented scenarios. The initial guesses for the states are drawn from the same bounds, and noise is introduced into the dynamics as a random acceleration with intensity $10^{-4}~g_{0}$. This value is compatible with the resolution provided by ZARM drop capsule’s IMU [25]. The mass and inertia tensor of each body are perturbed as well, sampling from a zero-mean normal distribution with standard deviation corresponding to the $5\%$ of their nominal value. To account for calibration uncertainty, the optical parameters are perturbed according to uniform distributions centered on their nominal values. A summary of the disturbances considered can be found in Table 13. Figure 15 shows the estimated positions and angular velocities for all converged samples in scenario 2, with the associated $3 \sigma $ ellipsoid. The estimation can be considered consistent, since only a number of estimates compatible with the $3 \sigma $ confidence level fall outside the ellipsoid computed by the optimizer. It can be noticed that the estimation of the angular velocity on the first arc exhibits a large variance. This outcome can be attributed to the fact that the nominal initial angular velocity is zero, meaning the motion is purely translational. As a result, the initial spin rate is poorly estimated. This suggests that imposing a non-zero initial angular velocity to the cobbles in the experiment will be beneficial from the estimation perspective. To compute $CoR_{E}$, the full trajectory is propagated taking the estimated state at $t^{1st}_{0}$ as the initial condition for each sample. Then, the energy at $t^{1st}_{f}$ is computed: this value corresponds to the energy before the collision. The post-collision energy is recovered from the estimated state at time $t^{2nd}_{0}$, and the $CoR_{E}$ can be computed applying Eq. (3). The sample mean and sample variance for the $CoR_{E}$ have been recovered, and are reported in Table 14, Table 15, and Table 16 for each case considered. The percentage of converged samples is reported as well. The estimation can be considered consistent, as the error lies within the $\pm 3 \sigma $ bounds. The outcomes of the statistical analysis for the $CoR_{E}$ estimation are reported in Fig. 16.

**Table 13:** Sampling ranges for Monte Carlo analysis

Disturbance	Distribution	Value
Intrinsic parameters	Uniform	± 10 px around nominal value
Translation vector	Uniform	Bounds ± 5 mm around nominal value
Rotation matrix	Uniform	Bounds ± 10^−4^ around nominal value
Distortion parameters	Uniform	Bounds ± 5% of nominal value
Inertia	Normal	$\sigma _{I} = 0.05 \cdot \max (I)$
Mass	Normal	$\sigma _{m} = 0.05 \cdot m$

**Table 14:** MC Analysis results, scenario 1

	% Converged	$CoR_{E} \ [-]$	$\sigma _{CoR_{E}} \ [-]$
$\sigma _{px} = 1~\mathrm{px}$	95	0.8458	0.0053
$\sigma _{px} = 2~\mathrm{px}$	90	0.8465	0.0070
$\sigma _{px} = 3~\mathrm{px}$	94	0.8464	0.0088

**Table 15:** MC Analysis results, scenario 2

	% Converged	$CoR_{E} \ [-]$	$\sigma _{CoR_{E}} \ [-]$
$\sigma _{px} = 1~\mathrm{px}$	97	0.8458	0.0051
$\sigma _{px} = 2~\mathrm{px}$	97	0.8453	0.0046
$\sigma _{px} = 3~\mathrm{px}$	98	0.8452	0.0089

**Table 16:** MC Analysis results, scenario 3

	% Converged	$CoR_{E} \ [-]$	$\sigma _{CoR_{E}} \ [-]$
$\sigma _{px} = 1~\mathrm{px}$	96	0.8455	0.0136
$\sigma _{px} = 2~\mathrm{px}$	94	0.8480	0.0124
$\sigma _{px} = 3~\mathrm{px}$	92	0.8429	0.0140

As a general remark it can be stated that placing cameras at the view-ports of the JAXA vacuum chamber, as implemented in scenario 1, provides accurate and consistent estimates of the cobbles’ states and the coefficient of restitution. Results obtained applying scenario 2 show that also placing one camera on the opposite side of the chamber is also a viable approach to get accurate estimates of states and parameters. Moreover, results relative to scenario 3 demonstrate that the loss of a camera leads to a drop in estimation performance. Although not dramatic, this suggests that redundancy is desirable in the camera network.

### Accuracy evaluation

**Fig. 17:**
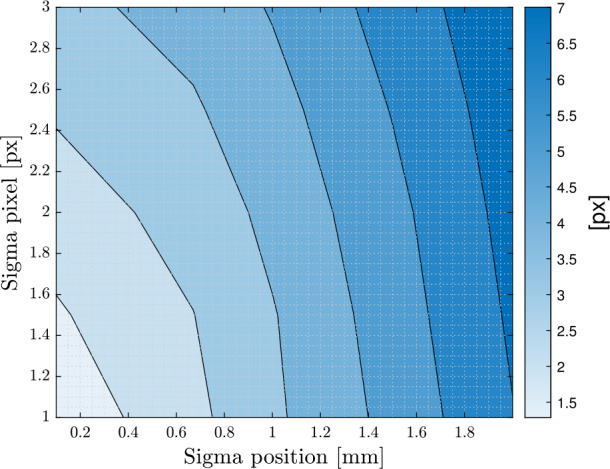
Average value of the post-fit residuals for varying levels of noise. Results relative to scenario 2

**Fig. 18:**
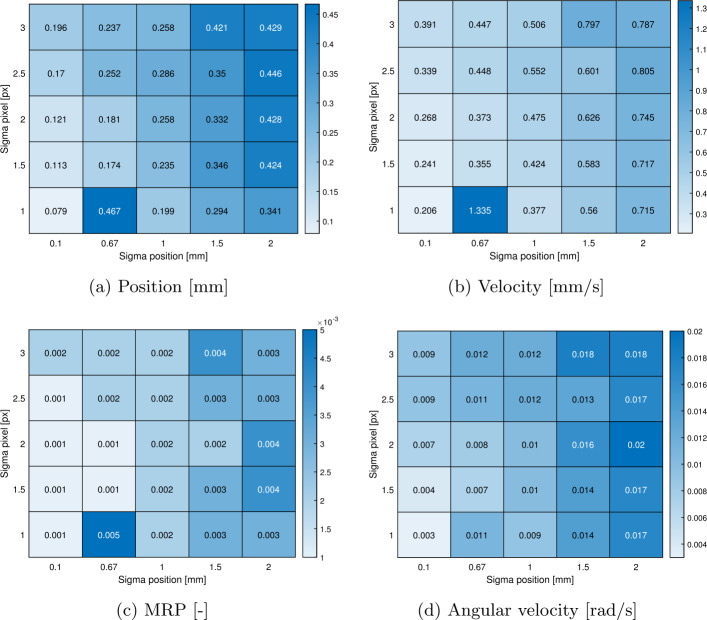
Heatmaps of standard deviation associated to the component of the states on second arc, body 1. Results from MC analysis relative to Scenario 2

This Section presents a method for evaluating the accuracy of the estimation in the real experiment. Given that no additional sensors will be available to validate the camera-based estimation, the accuracy achievable by the estimation algorithm is correlated with the average value of the post-fit residuals. These residuals are obtained by minimizing the cost function (Eq. (10), (11)), as proposed in [37]. To assess the estimation accuracy, 25 Monte Carlo simulations were conducted, employing various noise levels: $\sigma _{V} \in [0.1, 0.67, 1, 1.5, 2]$ mm and $\sigma _{px} \in [1, 1.5, 2, 2.5, 3]$ px. The contour plot of the average value of the post-fit residuals is shown in Fig. 17. The average value is chosen as a metric, as it is independent of the number of cameras and markers used in the estimation. As expected, the average residual obtained increases with the uncertainty in the tracking and markers’ position. Although a few outliers are present, the same trend is observed in the standard deviation of each state, as shown in Fig. 18. This procedure will also be applied to the experimental results. By comparing the average residuals derived from the real measurements with the values from the map, it will be possible to associate a standard deviation to the estimates of each state.

## Conclusions

The purpose of this paper is to present the concept, design and preliminary tests towards a micro-gravity experiment aimed at validating the contact dynamics of a multi-body code at particle scale. The overarching goal is to enhance the realism in complex asteroid dynamics simulations involving numerous particles. This is crucial for supporting future asteroid exploration missions and advancing our understanding of asteroid evolution processes from a scientific perspective. The experiment will be conducted at the ZARM Drop Tower and consists in observing the collision between two asteroid simulant cobbles using a set of cameras to reconstruct their full state trajectory. Following the experiment, a high-fidelity digital twin will be created in the N-body code GRAINS, which will be calibrated to reproduce the estimated trajectory with maximum accuracy. A benchmark model for the digital twin has been developed to generate a reference trajectory, which is used to test the state and contact parameter estimation procedure. The estimation strategy has been validated through numerical simulations across a range of scenarios, varying camera poses and noise levels in the measurements. Overall, the results demonstrate satisfactory performance in estimating the states and contact parameters. Hence, the preliminary validation campaign can be considered successful. Additionally, a methodology for evaluating the accuracy of the estimation procedure is proposed.

The experiment’s hardware has been designed to meet the constraints imposed by the ZARM facility. Key requirements for each component have been addressed and discussed. Moving forward, future work will focus on validating the estimation algorithm using real camera data, creating illumination conditions compatible with those present in the ZARM drop capsule, and assessing the reliability of the release mechanism through lab testing.

## Data Availability

No datasets were generated or analysed during the current study.
